# Giant Malignant Pheochromocytoma with Palpable Rib Metastases

**DOI:** 10.1155/2014/354687

**Published:** 2014-08-03

**Authors:** Esat Korgali, Gokce Dundar, Gokhan Gokce, Fatih Kilicli, Sahande Elagoz, Semih Ayan, Emin Yener Gultekin

**Affiliations:** ^1^Department of Urology, Cumhuriyet University Medical Faculty, 58140 Sivas, Turkey; ^2^Department of Endocrinology, Cumhuriyet University Medical Faculty, 58140 Sivas, Turkey; ^3^Department of Pathology, Cumhuriyet University Medical Faculty, 58140 Sivas, Turkey

## Abstract

Pheochromocytoma is a rare and usually benign neuroendocrine neoplasm. Only 10% of all these tumors are malignant and there are no definitive histological or cytological criteria of malignancy. Single malignancy criteria are the presence of advanced locoregional disease or metastases. We report a case, with a giant retroperitoneal tumor having multiple metastases including palpable rib metastases, who was diagnosed as a malignant pheochromocytoma. The patient was treated with surgery. The literature was reviewed to evaluate tumor features and current diagnostic and therapeutic approaches for patients with metastatic or potentially malignant pheochromocytoma.

## 1. Introduction

Pheochromocytoma is a rare neuroendocrine neoplasm that synthesizes, stores, metabolizes, and also secretes catecholamines. Pheochromocytoma has a prevalence rate of around 300,000 adults/year [1]. Having a poor prognosis, the incidence of metastatic pheochromocytoma is approximately 17% [2]. The histopathological evaluation cannot distinguish between benign and malignant tumors. In order to distinguish malignancy from multifocal disease, the only malignancy criterion is the presence of frank locoregional invasion or metastases at nonchromaffin sites which are distant from the primary neoplasm (such as liver, lungs, or bone) [3]. Several cases of giant pheochromocytoma with bone metastasis have been reported in the literature. We describe a malignant giant pheochromocytoma with multiple metastases which was presented as an acute coronary syndrome and palpable mass on the left side of the chest.

## 2. Case

**Figure 1:**
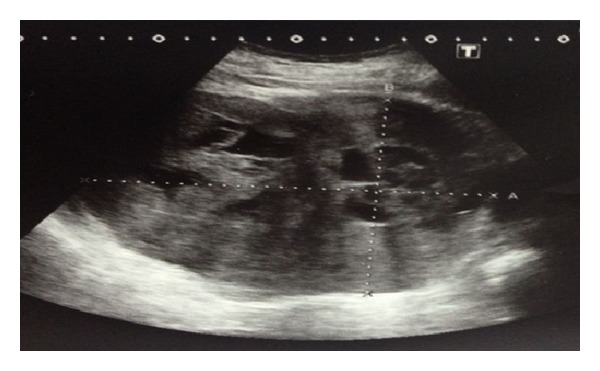
The 150 × 140 × 130 mm (RL × AP × KK) mass in left adrenal gland in abdominal ultrasonography.

**Figure 2:**
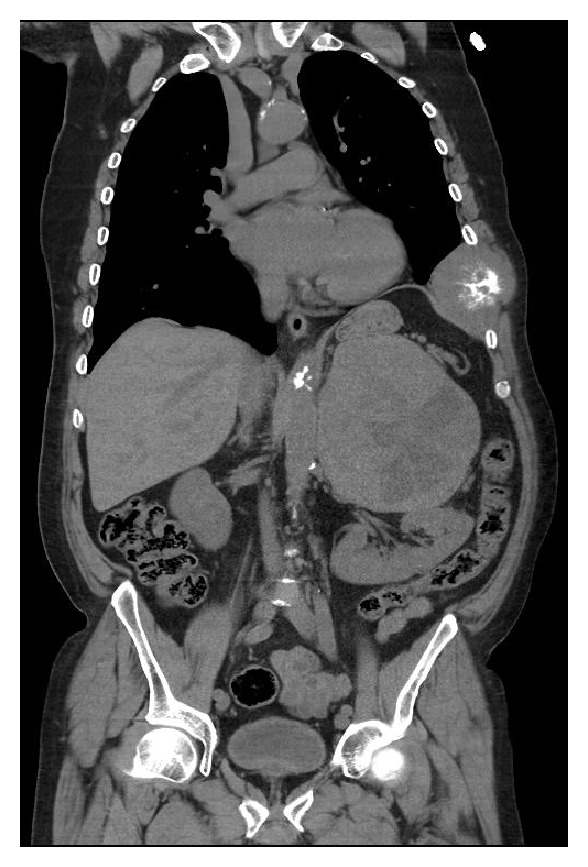
127 × 143 × 144 mm (RL × AP × KK) mass in the left adrenal surgical space, pushing down the left kidney on CT and the rib metastasis.

**Figure 3:**
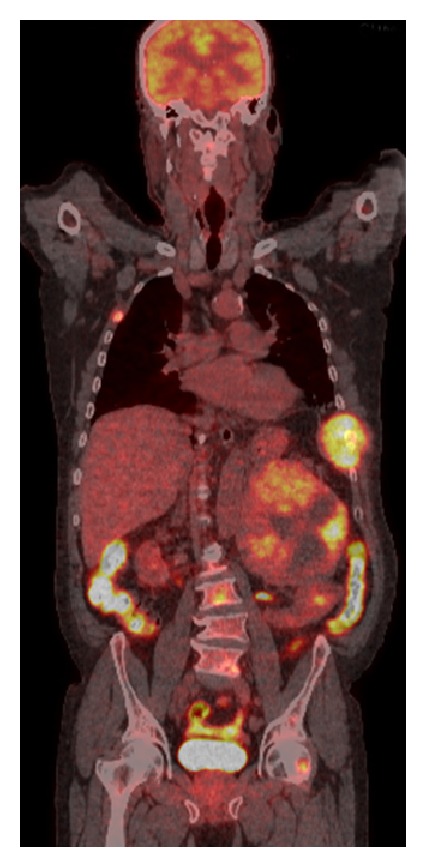
127 × 143 × 144 mm (RL × AP × KK) mass in the left adrenal with SUV max: 12.7 and widespread involvement of the skeletal system (multiple ribs, sacrum, sacroiliac area, and left femur).

**Figure 4:**
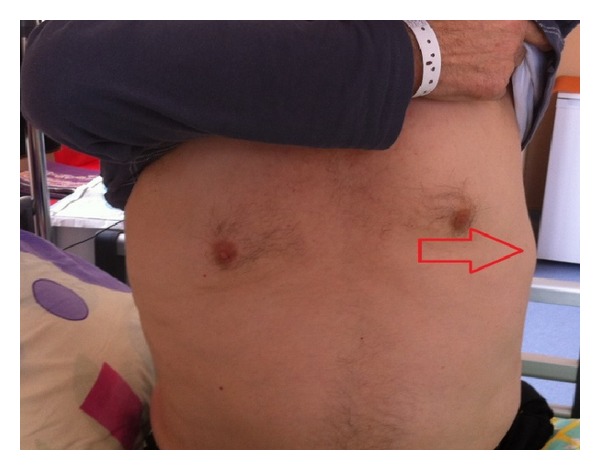
Thoracic inspection of patient.

**Figure 5:**
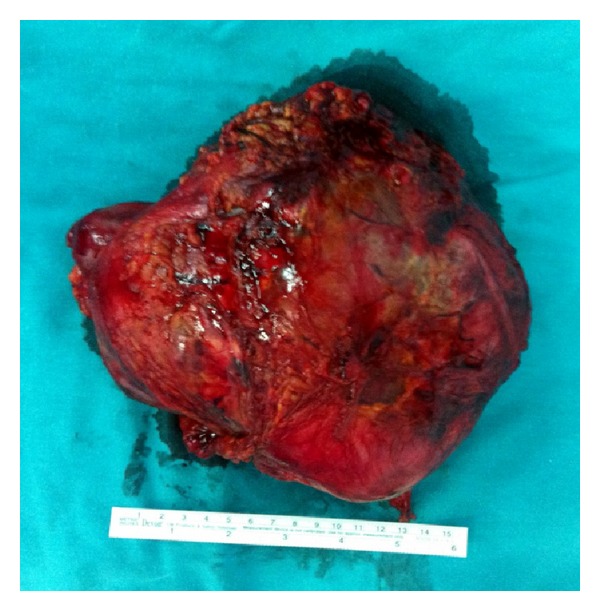
Macroscopic view of adrenal mass.

**Figure 6:**
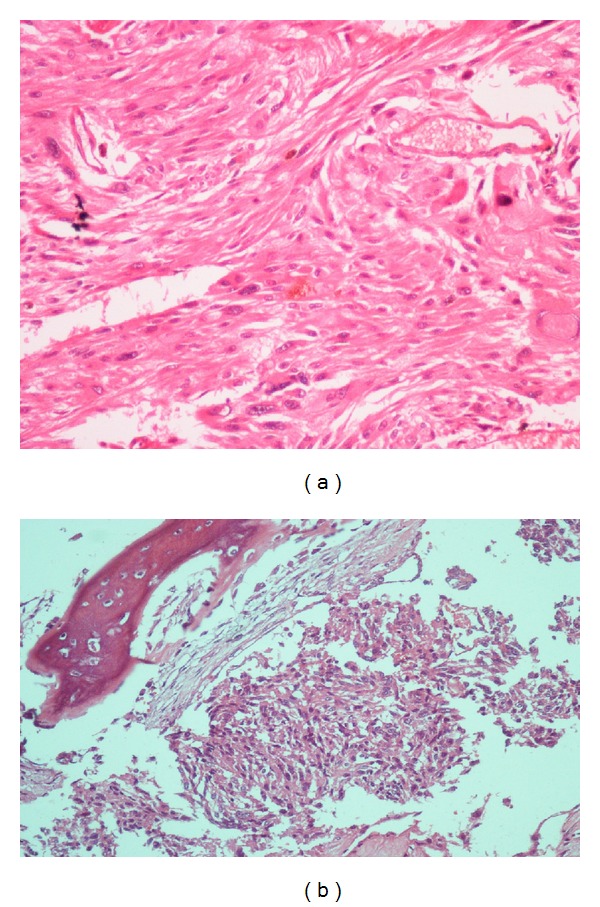
Pathological view. (a) Pleomorphic spindle-shaped tumor cells with the lost organoid pattern in surrenal gland histology (H&E × 400). (b) Tumoral cells infiltrating the bone with similar morphology of adrenal mass in rib histology (H&E × 200).

A case of 63-year-old man with complaints of severe chest pain, sweating, and nausea was investigated. Acute coronary syndrome was considered as the differential diagnosis, but the coronary angiography was normal. Abdominal ultrasound demonstrated a 15 × 14 × 13 cm mass in the left adrenal gland (Figure 1), and then patient was referred to our clinic. Physical examination of the patient revealed not only nonpainful, firm, and mobile abdominal mass in left hemi-upper abdomen but also painful mass on his left 8th, 9th, and 10th ribs (Figure 4). His blood pressure was 185/95 mmHg and pulse was 86 per minute. Thoracoabdominal computed tomography (CT) revealed 12.5 × 14.5 × 14 cm mass located in the left retroperitoneum. This mass appeared to be invading the left kidney and there were cystic areas and calcifications (Figure 2). Also both in CT scan and PET scan, multiple metastases in both lungs, right lobe posterior-inferior subsegments of liver, and the skeletal system were detected (Figure 3). The results of blood analysis (glucose, uric acid, plasmatic proteins, ions, and complete blood count) were normal. Plasma and urinary catecholamamines and metanephrines were elevated (Table 1). He received 4 mg doxazosin four times a day for 15 days prior to the surgery. Three days after blocking alpha, he also received 50 mg metoprolol twice a day and 20 mg amlodipine once a day for preoperative preparation. For 3 days prior to the operation, he received daily 3500 ml saline for hydration and blood pressure was titrated with Na-nitroprusside during the operation. During the surgical exploration with thorachoabdominal incision, 1736 grams of 20 × 17 × 9 cm fixed round well-vascularized retroperitoneal mass involving the surrenal gland (Figure 5) and the mass in ribs 7–10 were observed. Left surrenalectomy and excision of ribs which were surrounded with soft tissue mass were done without complication while high blood pressure attacks were normalized with Na-nitroprusside during the operation. There were no postoperative complications. Histological analysis showed a solid-cystic adrenal neuroendocrine tumor and pathology resulted as malignant pheochromocytoma (Figure 6) with rib metastasis.

## 3. Discussion

Pheochromocytomas are rare catecholamine-secreting tumors derived from the chromaffin cells of the embryonic neural crest. The triad of headache, sweating, and palpitations in patients with hypertension is diagnostic, with 94% specificity and 91% sensitivity [4]. Preoperative diagnosis is usually established by the presence of clinical signs and the determination of catecholamines and their metabolites in blood and urine. Malignant pheochromocytomas are rare and the diagnosis of malignancy is not primarily based on cytological characteristics but is defined by the presence of local invasion or metastatic disease like other neuroendocrine tumors and the most common locations of metastatic spread are the lymph ganglia, bones, liver, and lungs [2, 5]. Metastases may be synchronous or metachronous. The incidence of metastatic pheochromocytoma is approximately 17% and has a poor prognosis, with 40–74% chance of surviving for 5 years [2]. The peak age of occurrence is in the third to fifth decades of life except for familial forms [6]. Specific biochemical markers do not indicate malignancy in PHs and SPGs and the presence of metastases should be documented by using imaging tests. MRI or CT is always recommended to evaluate dissemination in the thoracic, abdominal, and pelvic cavities. Bone scanning is always recommended to rule out skeletal metastases [7]. Functional imaging studies such as I^123^ metaiodobenzylguanidine (MIBG) or F^18^-fluorodeoxyglucose positron emission tomography (FDG-PET) scans are also recommended because they may help to detect metastases that cannot be detected by CT or MRI [8]. Although FDG-PET is also likely the best diagnostic imaging modality to use for some patients with distinct sporadic metastatic pheochromocytomas and sympathetic paragangliomas, FDG-PET is not the best imaging modality to use for every patient with sporadic malignant disease. In fact, in some of these patients, metastatic lesions cannot be detected by using FDG-PET, and disease extension is best evaluated by other imaging modalities, such as MIBG [9].

Surgical resection is the only curative treatment for pheochromocytomas and sympathetic paragangliomas. Because there has been currently no effective cure for malignant pheochromocytoma, most treatments are palliative; however, in some cases, tumor and metastasis resection, if resectable, improve survival and quality of life and reduce exposure of the cardiovascular system [9]. In patients with noncurable disease, the goals of surgery are to reduce hormone secretion, prevent complications related to a critical anatomical location, and, perhaps, increase the efficacy of other therapies [9]. Before surgery, the patient must be adequately prepared with alpha- and beta-adrenergic blockade and a complete restoration of the fluid and electrolyte balance [10]. Without appropriate preoperative preparation, induction of anesthesia, tumor manipulation, or pharmacologic stimulation by opioids, antiemetics, neuromuscular blockage, vagolytics, or sympathomimetics could result in massive intraoperative outpouring of catecholamines, with subsequent hypertensive crisis and possible stroke, arrhythmia, or myocardial infarction [8]. Radiation therapy is also a useful option for the palliation in these cases (mainly in bone metastases). Current chemotherapy, cyclophosphamide-based and dacarbazine-based regimens combined with vincristine or doxorubicin are the best studied regimens and it can achieve partial remission and improvement of clinical symptoms in more than half of all cases; some patients even experience complete remission. Recently, some studies reported promising results of a therapy with targeted therapies in patients with malignant pheochromocytoma [11, 12]. Treating metastatic lesions by hormonal blocking with therapeutic doses of iodine-131MIBG gives good results in residual or irresectable disease [9]. Follow-up is also important and, with time, we can determine the malignant tumoral behavior.

Our case is a patient with a large-sized tumor (1750 gr) with multiple metastases in multiple organs which is denominated as malignancy. There were a large number of mitoses in the pathology evaluation and a metastatic rib masses which confirmed the diagnosis.

## 4. Conclusion

The giant symptomatic, secreting pheochromocytoma with multiple metastasis in multiple organs is rare. We would like to emphasize the importance of preoperative diagnosis and using imaging modalities that can establish the right diagnosis of malignant giant pheochromocytoma. The treatment in these forms requires a multidisciplinary management. In this manner, we can reduce the mortality and the complications with increased survival rates and improved quality of life of patients.

## Figures and Tables

**Table 1:** The values of some metabolites of our patient.

	Laboratory standard ranges	Preoperative value	Postoperative value
Metanephrine (24 h urine)	52–341 ug/day	29259 ug/day	109,25 ug/day
Normetanephrine (24 h urine)	88–444 ug/day	71731 ug/day	728,34 ug/day
Epinephrine (blood)	0–60 mg/day	829 mg/day	
Norepinephrine (blood)	120–680 mg/day	8384 mg/day	
Dopamine (blood)	0–87 pg/mL	250 pg/mL	
Dopamine (24 h urine)	65–400 ug/day	8420 ug/day	
Vanillylmandelic acid	3–9 mg/day	204,9 mg/day	
1 mg dexamethasone suppression		<1 ug/dL	
Plasma aldosterone (PA)/plasma renin activity (PRA)		<5	
